# Comparative Effectiveness Trial of an Obesity Prevention Intervention in EFNEP and SNAP-ED: Primary Outcomes

**DOI:** 10.3390/nu11051012

**Published:** 2019-05-05

**Authors:** Joanna Buscemi, Angela Odoms-Young, Melinda R. Stolley, Linda Schiffer, Lara Blumstein, Margaret H. Clark, Michael L. Berbaum, Jennifer McCaffrey, Carol Braunschweig, Marian L. Fitzgibbon

**Affiliations:** 1 DePaul University, Department of Psychology, 2219 N. Kenmore Ave, Chicago IL 60614, USA; mclark70@depaul.edu; 2 University of Illinois at Chicago, Institute for Health Research and Policy, 1747 West Roosevelt Road, Chicago, IL 60608, USA; lbb@uic.edu (L.B.); mberbaum@uic.edu (M.L.B.); mlf@uic.edu (M.L.F.); 3 University of Illinois at Chicago, Department of Kinesiology and Nutrition, 1919 West Taylor Street, Chicago, IL 60612, USA; odmyoung@gmail.com (A.O.-Y.); braunsch@uic.edu (C.B.); 4 Medical College of Wisconsin, General Internal Medicine, 8701 Watertown Plank Road, Milwaukee, WI 53226, USA; Mstolley@mcw.edu (M.R.S.); lschiff@uic.edu (L.S.); 5 Family and Consumer Sciences, University of Illinois Cooperative Extension Service, University of Illinois at Urbana-Champaign, 905 S. Goodwin Ave, Urbana, IL 61801, USA; jmccaffr@illinois.edu; 6 Department of Pediatrics, University of Illinois at Chicago, 840 S. Wood Street, Chicago, IL 60612, USA; 7 University of Illinois Cancer Center, University of Illinois at Chicago, 818 South Wolcott Avenue, Chicago, IL 60612, USA

**Keywords:** obesity prevention, preschool children, USDA, dissemination, EFNEP, SNAP-Ed

## Abstract

There is a need to disseminate evidence-based childhood obesity prevention interventions on a broader scale to reduce obesity-related disparities among underserved children. The purpose of this study was to test the comparative effectiveness of an evidence-based obesity prevention intervention, Hip-Hop to Health (HH), delivered through Expanded Food and Nutrition Education Program (EFNEP) and the Supplemental Nutrition Assistance Program-Education (SNAP-Ed) versus the standard curriculum delivered by the programs (Standard Nutrition Education (NE)). A nonequivalent control group design was delivered to compare the effectiveness of HH to NE on weight gain prevention and health behavior outcomes at EFNEP and SNAP-Ed sites. One hundred and fifty-three caregiver–child dyads (*n* = 103 in the HH group; *n* = 50 in the NE group) participated in the study. HH is an evidence-based dietary and physical activity intervention for low-income preschool children. The NE curriculum provided lessons for children that are consistent with the Dietary Guidelines for Americans 2010. Data were collected on demographics, anthropometrics, and behavioral variables for parent–child dyads at baseline and postintervention. Mixed model methods with random effects for site and participant were utilized. No differences in child or caregiver diet, physical activity, or screen time by group were found. No between-group differences in child BMI z-score were found; however, caregivers in the HH group lost significantly more weight than those in the NE group. Results from this trial can inform future dissemination efforts of evidenced-based programs for underserved families.

## 1. Introduction

Obesity is an epidemic in the US, [1,2,3] and increases risk for comorbidities such as heart disease, diabetes, and some cancers [4]. In 2016, 13.9% of US preschool-aged children were obese [5]. Patterns of obesogenic behavior initiated in early childhood are linked to behaviors in adulthood, and lead to poor health outcomes [6,7,8].

Rates of obesity are higher among minority children. In 2013–2016, 9.9% of Non-Hispanic White, 11.6% of Non-Hispanic Black, and 16.5% of Hispanic preschool children were obese. Additionally, prevalence rates increased by age; for example the prevalence rate of obesity was higher among 6–11 year olds (14% of Non-Hispanic Whites, 19.8% of Non-Hispanic Blacks, and 25.3% of Hispanics) [2] suggesting that children may be at increased risk of weight gain between early childhood and middle childhood, and these increases are even more pronounced in minority children [9]. Furthermore, children of low-income households are at risk for obesity and poor diet quality [10,11]. Therefore, the preschool years may represent an important point of intervention for addressing obesogenic health behaviors to prevent obesity long term, especially among low-income minority children [12,13,14]. 

Very few evidence-based obesity prevention interventions for low-income and minority children have been implemented and disseminated broadly [15,16,17]. The National Institutes of Health [18] have highlighted the need for research focused on the implementation and dissemination of evidence-based interventions [17]. “Hip-Hop to Health” (HH) is a school-based nutrition and physical activity obesity prevention program for low-income preschool children [13]. In a previous trial, the HH intervention was compared to a General Health Curriculum (GHC) in 3- to 5-year-olds in a predominantly African American sample and found that children randomized to receive the HH intervention had significantly smaller increases in Body Mass Index (BMI) *z*-scores compared to children in the GHC control group at 1- and 2-year follow-ups [13]. HH was the first intervention to exhibit positive effects on BMI *z*-scores in low-income, preschool children [13]. In several school-based follow-up trials utilizing the same basic curriculum but tailoring the content and/or interventionist, positive changes in preschool children’s obesogenic behavior for diet quality, screen time, and physical activity were found [13,19,20,21,22]. In each of these trials, however, barriers to parent participation were encountered, primarily due to competing priorities. Although parents did not receive an in person intervention, they were encouraged to participate through weekly homework assignments based on newsletters that mirrored the intervention classroom curriculum, and received $5 for each completed homework submission. In the original HH trial, 39% of parents did not complete any homework assignments [13], and in the effectiveness trial, parents only completed 5 of 13 assignments on average [21]. Therefore, there is a need to develop interventions that can also reach parents who are overburdened.

The Expanded Food and Nutrition Education Program (EFNEP) is a United States Department of Agriculture (USDA) funded program focused on nutrition, food safety, and food resource management education. EFNEP was developed primarily to improve dietary quality among low-income families. Also funded by the USDA, the Supplemental Nutrition Assistance Program Education (SNAP-Ed) aims to provide nutrition education to encourage Supplemental Nutrition Assistance Program (SNAP) recipients to make healthier food purchases consistent with the Dietary Guidelines for Americans [23]. Previous research has shown that these programs improve meal planning, comparison shopping, and diet [24,25]. Given these positive findings and the fact that low-income families frequent these programs, they may be an ideal setting for the implementation and dissemination of evidence-based obesity prevention interventions [26]. 

To address these barriers of widespread implementation and dissemination of HH and more fully engage parents, the current study aimed to test the comparative effectiveness of the adapted HH versus the standard curriculum delivered by the programs (Standard Nutrition Education (NE)). Our primary outcomes were changes in diet, physical activity, and screen time among both parents and children. Group differences in body mass index trajectories were also analyzed as an exploratory aim. It was hypothesized that postintervention, children and caregivers receiving the HH intervention would exhibit (1) improved dietary intake; (2) higher levels of physical activity; and 3) lower levels of screen time as compared to the NE participants.

## 2. Materials and Methods 

### 2.1. Design 

This Institutional Review Board approved comparative effectiveness study utilized a quasi-experimental nonequivalent control group design comparing HH to the usual EFNEP and SNAP-Ed NE curriculum for low-income parents and children. Nonequivalent control group designs are commonly used in implementation science research when a randomized design is not possible given the needs of the community, small sample size, or other logistical considerations [27]. In this case, some of the initial sites enrolled needed to begin their programs immediately after recruitment, leaving insufficient time to train the peer educators. Given this limitation, these initial sites became NE sites, and subsequent sites were HH sites. Parent–child dyads (one parent/child per household) in HH received an eight-lesson obesity prevention intervention that was delivered over 6–8 weeks. NE parent–child dyads received the standard curriculum, also delivered in 8 lessons over 6–8 weeks. Data were collected on weight, height, dietary intake, physical activity, and screen time at baseline and postintervention. In a few cases, the enrolled parent was unavailable postintervention, and another parent/guardian gave information about the child. This work was approved by the Institutional Review Board at the University of Illinois at Chicago (SDA IRB number is 2010–1004) and follows the guidelines of the Declaration of Helsinki.

#### 2.1.1. Sample 

One-hundred and fifty-three child–parent dyads were enrolled (*n* = 103 in the Intervention group; *n* = 50 in the NE group). Table 1 and Table 2 indicate the demographics of the enrolled children and caretakers. Although EFNEP and SNAP-Ed provide services in a variety of settings, participants were recruited primarily from preschools in order to efficiently recruit families and young children, and were approached to participate during pick-up and drop-off times. All children in each preschool classroom were invited to participate with their parents if they were 2–5 years old and recipients of EFNEP and SNAP-Ed services at the participating sites. Parents signed an informed consent for themselves and their children. Each parent was paid $25 for their participation upon completion of the interview and at postintervention for a total of $50. 

#### 2.1.2. Measures 

Data were collected at baseline and immediately at postintervention. All measures were administered at both visits unless otherwise specified. Given the goals of the study, which included ease of dissemination and sustainability for the community, brief and simple measures were utilized. As such, quick, self-report data provided by parents on behalf of their child were selected. 

#### 2.1.3. Demographics

Parents reported date of birth (own and child), sex (own and child), race-ethnicity (own and child), relationship to the child, education, employment, marital status, country of birth, car ownership, and public assistance in the last six months (Women, Infants and Children (WIC), SNAP, cash assistance) at baseline.

#### 2.1.4. Anthropometrics

Children’s and caregivers’ height and weight were measured at baseline and postintervention in light clothing and without shoes. Weight was measured using a digital scale, and height was measured using a portable stadiometer. BMI was calculated as weight in kilograms divided by the square of height in meters. Child BMI *Z-*scores and BMI percentiles for age and sex were calculated for each child based on the 2000 Centers for Disease Control and Prevention (CDC) Growth Charts [28].

#### 2.1.5. Dietary Intake

Starting the Conversation (STC) is a brief, eight-item measure designed for clinical assessment of dietary habits and patterns [29]. The measure is robust across demographic variables, including health literacy, gender, and education-level, and has strong test-retest reliability (*r* = 0.66). STC survey items are measured on a three-point Likert scale: the most healthful dietary practices (e.g., fast food less than one time per week, five or more servings of fruit per day), moderately healthful practices (e.g., fast food 1–3 times per week, 3–4 servings of fruit per day), and least healthful practices (e.g., fast food four or more times per week; two or fewer servings of fruit per day). Scores are summed and range from 0–16, with higher values reflecting a high-quality diet. 

#### 2.1.6. Physical Activity

The Godin Leisure-Time Exercise Questionnaire assesses caregiver report of how many times they or their child engage in strenuous exercise, moderate exercise, or mild exercise during their free time for more than 15 min at a time during a typical seven-day period [30]. The children’s questionnaire specified that free time does not include time at school. Examples of activities were also revised to be appropriate for children, and included activities such as running, gymnastics, soccer, roller skating, soccer, jumping rope, dancing, and playing around the house. A weekly activity score was calculated from the responses: 9 × Strenuous + 5 × Moderate + 3 × Light.

#### 2.1.7. Screen Time

Caregivers were asked to estimate the number of hours their child spends on a typical school day (or a typical weekday if the child is not in school) and on a typical weekend day; (1) watching television (broadcast, cable, or satellite); (2) watching DVDs or videos; (3) playing video games while sitting down (not including active video games); or (4) using a computer. Caregivers also reported their own screen time on a typical weekday and on a typical weekend day. Screen time (hours/day) was calculated as follows: (5 × (all screen time on an average weekday) + 2 × (all screen time on an average weekend day))/7 [31].

### 2.2. Intervention

#### 2.2.1. Adaptation of HH for Children and Parents 

The original HH was adapted in collaboration with our EFNEP and SNAP-Ed partners and other stakeholders (e.g., parents of preschoolers, USDA staff, etc.) The adapted HH curriculum utilized the most salient lessons and activities from the original HH to fit the needs of EFNEP and SNAP-Ed for a flexible curriculum. The instructor (one per site) was able to tailor the curriculum in order to accommodate any constraints related to time and resources. The study team decreased the number of sessions from three times per week for 14 weeks to one time per week for 6–8 weeks to be consistent with the format of EFNEP and SNAP-Ed and to promote intervention adherence. 

The parent curriculum was developed based on the principles of child feeding guidelines, recommendations for physical activity and limiting screen time, and the previous parent newsletters that were developed in Hip-Hop to Health [19]. 

#### 2.2.2. Standard Nutrition Education Intervention 

Participants in the NE group received the standard EFNEP and SNAP-Ed lessons they routinely provide for children that are consistent with the Dietary Guidelines for Americans 2010 [23]. The number of lessons, dose, delivery, and target population were identical to the HH condition. However, the content differed. Specifically, the parent curriculum utilized a combination of dialogue and “hands-on” activities to encourage behavior change related to making healthy food choices, food safety, food budgeting, and physical activity. Additionally, the NE program was developed for older children (5 years and older), did not have a complementary parent and child curriculum, and did not have an explicit focus on weight gain prevention. More details regarding the intervention content have been reported elsewhere [27].

#### 2.2.3. Training 

Twenty-three nutrition educators were trained on the adapted HH curriculum; training lasted approximately 12 h over the course of 2–3 days. 

### 2.3. Analysis

To test for differences between groups at baseline, t-test with pooled variance, Wilcoxon rank sum tests, chi-square tests, and Fisher’s exact tests were utilized. To test the effects of the intervention on the outcome measures, random intercept mixed models in SAS PROC MIXED were utilized, with random effects for site and participant and the baseline value included in the outcome vector: y = b_0_ + b_1_group + b_2_visit + b_3_group × visit. These models were run with and without adjustment for age, sex, race (white, other), parent’s education (<High School, High School graduate, Bachelor’s degree), parent’s employment (full- or part-time, other), parent’s marital status (married or living with partner, other), and SNAP participation in the last 6 months. All statistical analyses were performed using SAS v9.4 (SAS Institute, Cary, NC, USA). 

## 3. Results

The HH and NE groups had somewhat different demographics, with significant differences in child (Table 1) and parental (Table 2) age and race, parental education, employment and car ownership, and public assistance in the past six months. However, there were no significant differences between groups in diet, physical activity, or BMI, though HH children did have more screen time at baseline: 4.3 vs. 3.3 hours (h)/day, *p* = 0.02.

**Figure 1:**
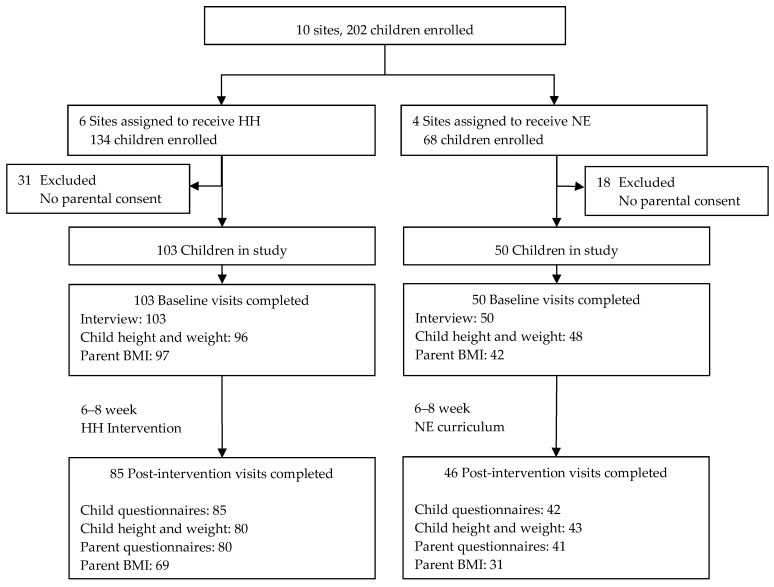
Flow of participants throughout the study.

Figure 1 describes the flow of participants through the study. Retention postintervention was 86% for children (83% in the Intervention group and 92% in the NE group) and 79% for parents (78% in the Intervention group and 82% in the NE group). 

Table 3 shows the estimated mean changes from baseline to postintervention in the HH and NE groups. There were no differences in the child BMI z-score mean change by group (*p* = 0.27). However, there were small but statistically significant differences in parent BMI change by group (HH mean change = −0.20 kg/m^2^; *SE* = 0.10; NE mean change = 0.29 kg/m^2^; *SE* = 0.15; *p* = 0.007). There were no significant differences between groups in children’s diet (*p* = 0.76), physical activity (*p* = 0.13) or screen time (*p* = 0.31).

## 4. Discussion

The current trial sought to adapt HH, an evidence-based obesity prevention intervention for underserved children, and deliver it in EFNEP and SNAP-Ed programs. We were successful in adapting the HH curriculum and training peer educators, and these peer educators were successful at delivering HH in EFNEP and SNAP-Ed. We fully involved our stakeholders in all aspects of the project and provided extensive training for our peer educators. We also tested the comparative effectiveness of the adapted HH intervention versus the standard NE curriculum delivered by the programs on our primary outcomes, namely, changes in diet, physical activity, and screen time among both parents and children. At postintervention there were no differences in the child BMI *z*-score mean change by groups. However, there were small, but statistically significant differences in parent BMI by group. This finding suggests that the original NE program delivers strong education, but that the integrated HH intervention may have additional benefits for parents. Previous research has demonstrated that parent weight changes often serve as an independent predictor of child weight changes [32]. Therefore, it was somewhat unexpected that we did not find differences in child weight. However, it is possible that the weight lost by caregivers was not significant enough to have an impact on child weight. It is also possible that our follow up was not long enough to detect the influence caregiver weight loss may have had on the child. Despite differences in parent BMI, surprisingly, no between group differences were found on parent health behaviors. This could be due to lack of sensitivity in measurement in terms of diet, physical activity, and screen time. 

Our findings should be interpreted in the context of some limitations. It is possible that the relatively short duration of the program (6–8 weeks) was insufficient to bring about significant changes in most of our outcomes. Similarly, the study team decreased the number of sessions from the original three times per week for 14 weeks to one time per week for 6–8 weeks to be consistent with the format of EFNEP and SNAP-Ed and to promote intervention adherence. However, even though they were small, the statistically significant differences in parent BMI outcomes from the HH group are promising given that this was the initial testing of our adapted parent curriculum. Future studies may increase the dose of the intervention and reassess outcomes longer-term (e.g., three months and six months postintervention). We were unable to utilize gold standard measurement for physical activity (accelerometry) and diet (dietary recall). Indeed, previous studies testing the efficacy of the EFNEP NE program on dietary outcomes conducted dietary recalls with participants [24,25]. Both studies found improvements in dietary outcomes among participants. We selected our measures specifically to enhance ease of dissemination, consistent with the goals of the study. Thus, we chose brief, relatively simple measures that could be utilized for future evaluation purposes. However, it is possible that the measures selected were not sufficiently precise to detect short-term changes in our outcomes. Additionally, we were unable to randomize our groups to treatment as initially planned. Instead, we utilized a quasi-experimental nonequivalent control group design comparing HH to NE, as some of the initial sites we enrolled needed to begin their programs immediately. Future studies should utilize a larger randomized design with a more equal number of participants in each group. Finally, there were some demographic differences between groups at baseline. Given these differences, we controlled for these variables as covariates in our analyses (see Table 3). 

## 5. Conclusions

Despite its limitations, this study was an important first step toward the dissemination of an evidence-based obesity prevention intervention for low-income and minority children. We adapted the HH curriculum for delivery in already-existing government funded community programs. We also developed a complementary parent curriculum. Finally, we trained peer educators to deliver this curriculum rather than research staff. Our research team continues to train SNAP-Ed facilitators to deliver HH in the context of their regular meetings, and the HH curriculum is now a part of the SNAP-Ed Toolkit: https://snapedtoolkit.org/interventions/programs/hip-hop-to-health-jr/. The full curriculum for HH can be found at https://www.ihrp.uic.edu/files/HHTH-curriculum-1aug2016.pdf. Future studies will test our curriculum within a randomized controlled trial. 

## Figures and Tables

**Table 1:** Children’s characteristics at baseline, by curriculum.

Variable Name	HH (Hip Hop) *N* = 103		NE (Nutrition Education) *N* = 50			All *N* = 153 ^a^	
	Mean or %	SD or N	Mean or %	SD or N	*P* ^b^	Mean or %	SD or N
Age, months	54.8	7.4	51.5	10.2	0.03	53.7	8.5
Sex					0.88		
Male	45%	46	46%	23		45%	69
Female	55%	57	54%	27		55%	84
Race					<0.001		
Hispanic/Latino	50%	52	6%	3		36%	55
African-American/Black, not Hispanic	33%	34	46%	23		37%	57
White, not Hispanic	10%	10	30%	15		16%	25
Multiracial, other	7%	7	18%	9		10%	16
BMI, kg/m^2^	16.9	2.0	16.5	1.9	0.26	16.7	1.9
BMI *Z* score	0.8	1.2	0.5	1.2	0.18	0.7	1.2
BMI percentile					0.29		
<5th (underweight)	4%	4	4%	2		4%	6
5th–<85th (normal weight)	51%	49	63%	30		55%	79
85th–<95th (overweight)	26%	25	17%	8		23%	33
≥95th (obese)	19%	18	17%	8		18%	26
Weight, kg	18.8	3.5	18.5	3.3	0.62	18.7	3.4
Height, cm	105.4	6.1	105.9	6.8	0.66	105.5	6.3
TV, h/day	2.4	1.5	1.9	1.5	0.04	2.2	1.5
Screen time, h/day	4.3	2.7	3.3	2.1	0.02	4.0	2.5
STC diet score (0–16) ^c^	9.6	2.2	9.9	2.1	0.47	9.7	2.2
Godin Leisure-Time Exercise ^d^	75.8	35.5	74.1	45.2	0.49	75.2	38.8
Godin Leisure-Time Exercise, moderate and strenuous only ^d^	61.0	32.4	58.2	41.6	0.32	60.1	35.5

^a^*N* = 144 for BMI; *N* = 149 for TV and screen time; *N* = 151 for Godin PA. ^b^ From *t*-tests with pooled variance, Wilcoxon rank sum tests, or chi-square tests for differences between groups at baseline. ^c^ A higher score indicates a better diet. ^d^ A higher score indicates more physical activity.

**Table 2:** Parents’ characteristics at baseline, by curriculum.

Variable Name	HH *N* = 103		NE *N* = 50			All *N* = 153 ^a^	
	Mean or %	SD or N	Mean or %	SD or N	*P* ^b^	Mean or %	SD or N
Age, years	32.9	8.6	37.5	9.6	0.004	34.4	9.2
Sex					0.31		
Male	14%	14	8%	4		12%	18
Female	86%	89	92%	46		88%	135
Relationship to child					0.69		
Mother	80%	82	84%	42		81%	124
Father	13%	13	8%	4		11%	17
Other	8%	8	8%	4		8%	12
Race					<0.001		
Hispanic/Latino	51%	53	6%	3		37%	56
African-American/Black, not Hispanic	34%	35	48%	24		39%	59
White, not Hispanic	11%	11	34%	17		18%	28
Multiracial, other	4%	4	12%	6		7%	10
Born in US (Hispanic only)	25%	13	67%	2	--^g^	27%	15
Years in US (if born outside US)	13.5	8.9	1.0	--	--^g^	13.1	9.0
Education, years	12.6	2.4	15.7	2.3	<0.001	13.6	2.8
Education, categories					<0.001		
Not HS graduate	21%	22	2%	1		15%	23
HS graduate/General Education Diploma	36%	37	10%	5		27%	42
Some college, no degree	19%	20	14%	7		18%	27
Associate’s degree	8%	8	4%	2		7%	10
Bachelor’s degree	16%	16	70%	35		33%	51
Employed full or part-time	36%	37	62%	31	0.002	44%	68
Marital status					0.17		
Single, never married	36%	37	28%	14		33%	51
Married or living with partner	56%	58	70%	35		61%	93
Separated, divorced, widowed	8%	8	2%	1		6%	9
Owns or leases car	47	47%	82%	41	<0.001	59%	88
Public assistance in last 6 months							
Cash assistance	8%	8	4%	2	0.50	7%	10
SNAP	52%	53	22%	11	<0.001	42%	64
WIC	30%	30	10%	5	0.006	23%	35
BMI, kg/m^2^	29.3	6.9	29.6	7.9	0.81	29.4	7.2
BMI category, kg/m^2^					0.96		
<18.5 (underweight)	0%	0	2%	1		1%	1
18.5–<25 (normal weight)	32%	31	26%	11		30%	42
25–<30 (overweight)	29%	28	33%	14		30%	42
≥30 (obese)	39%	38	38%	16		39%	54
Weight, kg	75.6	21.4	80.4	22.0	0.23	77.0	21.6
Height, cm	160.3	9.8	164.7	6.3	0.009	161.6	9.1
TV, h/day	2.8	2.1	1.9	1.3	0.02	2.5	1.9
Screen time, h/day	5.6	4.4	6.1	3.3	0.09	5.8	4.1
STC diet score (0–16) ^c^	9.5	2.9	9.7	2.8	0.82	9.5	2.9
Godin Leisure-Time Exercise score ^d^	52.2	44.9	46.7	32.8	0.64	50.4	41.3
Godin PA score, moderate and strenuous only ^d^	39.1	39.5	33.4	30.1	0.55	37.2	36.7

^a^*N* = 150 for age, car ownership, cash assistance, WIC; *N* = 152 for SNAP and Godin PA; *N* = 139 for BMI. ^b^ From *t*-tests with pooled variance, Wilcoxon rank sum tests, chi-square tests, or Fisher’s exact tests for difference between groups at baseline. ^c^ A higher score indicates a better diet. ^d^ A higher score indicates more physical activity.

**Table 3:** Estimated mean change from baseline to postintervention (6–8 weeks): Children and Parents.

Variable Name	No Covariates ^a^					Covariates ^b^				
	HH		NE			HH		NE		
	Mean Change	SD	Mean Change	SD	*P*	Mean Change	SD	Mean Change	SD	*P*
Children ^c^										
STC diet score (0–16) ^d^	0.34	0.24	0.18	0.34	0.71	0.29	0.24	0.16	0.34	0.76
Godin Leisure-Time Exercise ^e^	6.66	4.73	18.53 *	6.78	0.15	6.83	4.78	19.10 *	6.82	0.14
Godin Leisure-Time Exercise, moderate and strenuous only ^e^	4.39	4.23	15.39 *	6.07	0.14	4.72	4.28	16.00 *	6.11	0.13
TV, h/day	0.01	0.19	−0.36	0.27	0.25	0.04	0.19	−0.35	0.27	0.24
Screen time, h/day	0.20	0.23	−0.22	0.33	0.30	0.22	0.23	−0.19	0.33	0.31
BMI, kg/m^2^	0.00	0.04	0.10 *	0.05	0.11	0.00	0.06	0.11	0.07	0.23
BMI *Z* score	0.00	0.04	0.07	0.05	0.29	0.00	0.04	0.07	0.05	0.27
Parents ^c^										
STC diet score (0–16) ^d^	0.75 *	0.26	0.35	0.36	0.36	0.79 *	0.25	0.41	0.35	0.38
Godin Leisure-Time Exercise ^e^	3.01	4.67	6.99	6.57	0.62	3.50	4.77	6.74	6.70	0.69
Godin Leisure-Time Exercise, moderate and strenuous only ^e^	2.14	4.23	5.36	5.94	0.66	2.68	4.32	5.39	6.07	0.72
TV, h/day	−0.18	0.16	−0.19	0.22	0.96	−0.19	0.16	−0.19	0.22	0.98
Screen time, h/day	0.32	0.32	0.03	0.44	0.60	0.34	0.32	−0.03	0.45	0.51
BMI, kg/m^2^	−0.22 *	0.10	0.30	0.15	0.005	−0.20 *	0.10	0.29	0.15	0.007
Weight, kg	−0.59 *	0.28	0.79	0.42	0.008	−0.55	0.28	0.78	0.42	0.01

^a^ From random intercept mixed models with no covariates and random effects for site and participant: y = b_0_ + b_1_group + b_2_visit + b_3_group × visit. *P*-values are from tests for differences between groups in estimated mean change from baseline (the group × visit parameter). ^b^ From random intercept mixed models adjusted for age, sex, race (white, other), parent’s education (<HS, HS graduate, Bachelor’s degree), parent’s employment (full- or part-time, other), parent’s marital status (married or living with partner, other), and SNAP in last 6 months. ^c^ Baseline: *N* = 153; postintervention: *N* = 131 for children and 121 for parents. For child BMI, baseline N = 144, postintervention *N* = 123. For parent BMI, baseline = 139, postintervention *N* = 100. ^d^ A higher score indicates a better diet. ^e^ A higher score indicates more physical activity. * indicates a *p*-value of less than 0.05.
